# Thrombin-cleaved syndecan-3/-4 ectodomain fragments mediate endothelial barrier dysfunction

**DOI:** 10.1371/journal.pone.0214737

**Published:** 2019-05-15

**Authors:** Melanie Jannaway, Xiaoyuan Yang, Jamie E. Meegan, Danielle C. Coleman, Sarah Y. Yuan

**Affiliations:** 1 Department of Molecular Pharmacology and Physiology, Morsani College of Medicine, University of South Florida, Tampa, Florida, United States of America; 2 Department of Surgery, Morsani College of Medicine, University of South Florida, Tampa, Florida, United States of America; University of Illinois at Chicago, UNITED STATES

## Abstract

**Objective:**

The endothelial glycocalyx constitutes part of the endothelial barrier but its degradation leaves endothelial cells exposed to transmigrating cells and circulating mediators that can damage the barrier or promote intercellular gaps. Syndecan proteins are key components of the endothelial glycocalyx and are shed during disease states where expression and activity of proteases such as thrombin are elevated. We tested the ability of thrombin to cleave the ectodomains of syndecans and whether the products could act directly on endothelial cells to alter barrier function.

**Approach and results:**

Using transmission electron microscopy, we illustrated the presence of glycocalyx in human lung microvasculature. We confirmed expression of all syndecan subtypes on the endothelial surface of agarose-inflated human lungs. ELISA and western blot analysis suggested that thrombin can cleave syndecan-3/-4 ectodomains to produce fragments. *In vivo*, syndecan-3 ectodomain fragments increased extravasation of albumin-bound Evans blue in mouse lung, indicative of plasma protein leakage into the surrounding tissue. Syndecan-3/-4 ectodomain fragments decreased transendothelial electrical resistance, a measure of cell-cell adhesive barrier integrity, in a manner sensitive to a Rho kinase inhibitor. These effects were independent of glycosylation and thrombin receptor PAR1. Moreover, these cleavage products caused rapid VE-cadherin-based adherens junction disorganization and increased F-actin stress fibers, supporting their direct effect on endothelial paracellular permeability.

**Conclusions:**

We suggest that thrombin can cleave syndecan-3/4 ectodomain into fragments which interact with endothelial cells causing paracellular hyperpermeability. This may have important implications in the pathogenesis of vascular dysfunction during sepsis or thrombotic disease states where thrombin is activated.

## Introduction

The endothelial glycocalyx (eGCX) forms a protective luminal barrier composed of glycoproteins, glycolipids, proteoglycans and glycosaminoglycans. Providing functions essential to maintaining vascular homeostasis, the eGCX limits both vascular permeability and leukocyte-endothelial adhesion [1]. The type-1 transmembrane proteins, known as syndecans (SDC), are a family of four proteoglycans constituting a major component of the eGCX, and are constitutively shed from the endothelium typically as a result of direct action by different proteases.

A disintegrin-like and metalloprotease with thrombospondin type I motifs (ADAMTS1) and ADAMTS4 were both found capable of cleaving the SDC-4 ectodomain (S4ED) at a position near the N terminal [2]. Furthermore, the shedding of S4ED by ADAMTS1 resulted in cellular responses such as inhibition of migration, changes in F-actin distribution that correlated with cell rounding and a reduction in the number of focal adhesions. MMP14 (also called MT1-MMP), MMP2, MMP7 and MMP9 all have cleavage sites within the ectodomains of SDC-1 and SDC-4, whilst MMP3 can also cleave S1ED at a single site [3]. Recently, S2ED was also shown to be cleaved by MMP14 to produce multiple fragments. Moreover, the authors found that in cleaving S2ED, MMP14 impaired the ability of S2ED to decrease endothelial capillary-tube formation *in vitro*, which has implications in angiogenesis [4].

Evidence for the cleavage of S4ED by thrombin and plasmin, serine proteases involved in the coagulation cascade and the fibrinolytic system respectively, was shown when the substrate and enzymes were co-incubated *in vitro* [5]. The authors identified cleavage sites within the S4ED for these two circulating proteases. Plasmin cleaves the S4ED at Lys^114^ –Arg^115^ and Lys^129^ –Val^130^, whilst thrombin was shown to cleave at Lys^114^ –Arg^115^. A separate study found the same thrombin cleavage site within S4ED, as well as an additional site at Arg36-Tyr37 [3].

Multiple studies have shown that chronic inflammatory diseases such as sepsis, acute respiratory distress syndrome (ARDS) and acute lung injury (ALI) are associated with increased shedding of SDC ectodomains and related GCX components. Importantly, these diseases also exhibit increased levels and activity of many of the aforementioned proteases. Plasma concentrations of chondroitin sulfate, heparan sulfate, hyaluronic acid and S1ED were all increased in trauma patients compared to healthy controls [6], whilst S1ED serum levels in trauma patients have also been associated with increased mortality, inflammation and coagulopathy [7,8]. Murphy *et al*. (2017) measured increased circulating levels of S1ED in sepsis patients, and further showed that in patients with non-pulmonary sepsis, S1ED levels was associated with ARDS [9]. A recent study investigating plasma levels of all four syndecan members in intensive care unit patients, including sepsis and trauma, found increased levels of S1ED and S3ED compared to healthy controls; S2ED and S4ED were not different [10]. Disease conditions such as these can be further complicated by disseminated intravascular coagulation (DIC) [11], a disorder whereby systemic intravascular coagulation results in the production of microthrombi which can block the vasculature and restrict blood supply to organs [12]. Simultaneously the body is exhausted of available platelets thus uncontrolled bleeding also occurs.

Increased inflammation and coagulation abnormalities in vascular diseases and thrombotic conditions are closely linked with vascular barrier injury. Uncontrolled vascular permeability leads to tissue edema, particularly within the lungs [13,14]. In our study, we hypothesize that the serine protease thrombin, an enzyme persistently overproduced during DIC, can cleave syndecan ectodomains, either directly from the luminal surface of the endothelium or those already in circulation, to generate syndecan ectodomain fragments. We further hypothesize that these fragments may, in turn, act on the endothelium as signaling enhancers of barrier dysfunction. Our studies focus particularly on the endothelial glycocalyx within the pulmonary vasculature, visualizing glycocalyx and syndecan expression on *ex vivo* human lung endothelium, and the effects of their degradation products on endothelial barrier integrity.

## Material and methods

### Ethical approval

C57BL/6J mice (2 male and 2 female per group) were used aged 16–17 weeks and between weights 22-33g. Mice were maintained under a 12/12-hour light/dark cycle with food and water *ad libitum*. The Institutional Animal Care and Use Committee (IACUC) at the University of South Florida approved all surgical and experimental procedures (protocol number: IS00004121). The University of South Florida operates under the American Association for Laboratory Animal Science.

### Reagents

The source for all reagents is listed in S1 Table.

### Human lungs

Intact, viable lungs were provided by LifeLink, a Tampa-based nonprofit corporation that operates federally certified organ procurement organizations and FDA/AATB certified tissue banks to recover and process human organs/tissues for transplantation. An MTA has been executed enabling transfer of non-transplantable organs for research at the University of South Florida. Lungs were surgically removed by designated surgeons, processed according to the standard transplant protocols, and transported via authorized medical carriers. Lungs were received at the laboratory *en bloc* having been flushed with static preservation solution (SPS-1)/UW Solution and partially inflated before being closed at the trachea. Typical time from cross clamp to excision was 45–60 minutes.

### Human lung slices for immunohistochemistry

For sectioning of slices on the vibratome, the left upper lobe was isolated, and the airways filled with 3% w/v agarose in Dulbecco’s Modified Eagle Media (DMEM) via the bronchus. The lobe was placed on ice and the agarose allowed to set for 30 minutes before small sections of the lobe were cut and prepared for vibratome sectioning. Sections (200 μm thick) were cut on a vibratome in Hanks balanced salt solution. Sections were then fixed for 24 hours in 10% neutral buffered formalin, before being blocked and permeabilized with 10% donkey serum, 0.1% triton X, 0.1% tween in PBS for 60 minutes. Sections were stained with DAPI hydrochloride (10 μg/ml) as well as primary antibodies for CD31/PECAM-1 (1:250) and either SDC-1 (1:200), SDC-2 (1:100,), SDC-3 (1:100), SDC-4 (1:100) for 2 hours at room temperature. Sections were washed 5 x 5 minutes in 0.1% tween in PBS, then incubated for 2 hours at room temperature with AF488-conjugated donkey anti-mouse (1:500) and AF647-conjugated goat anti-Armenian hamster (1:250) secondary antibodies. Following 5 x 5-minute washes in 0.1% tween in PBS, the sections were mounted on slides with ProLong diamond antifade mountant. Images were taken using an Olympus FV1200 spectral inverted laser scanning confocal microscope with associated Olympus FluoView software. Approximately 15 slices were taken and used to create a maximum projection Z-stack.

### Human lung slices for supernatant collection

For collection of supernatant, the left upper lobe was cut into sections measuring 2 x 2 x 1 cm and weighing approximately 1.5g. Sections were incubated overnight at 37°C/5% CO_2_ in DMEM, before being treated with thrombin (10 U/ml) or vehicle for 24 hours, after which the supernatant was collected and frozen for future use in ELISAs.

### Enzyme-linked immunosorbent assay

ELISAs to determine the concentration of S1ED, S2ED, S3ED and S4ED in the human lung slice supernatant were carried out according to manufacturer’s instructions.

### Transmission electron microscopy

Human lung fragments were fixed with 3.5% glutaraldehyde, 0.08% Alcian Blue 8GX, 1% lanthanum nitrate hexahydrate in 0.1 M sodium cacodylate for 24 hours at 4°C, before being transferred to 0.1 M sodium cacodylate. The samples were embedded in resin and sectioned at 70–100 nm. Electron micrographs were taken using a JEOL JEM1400 transmission electron microscope with a side mounted digital Gatan 832 Orius camera. The associated software used was Digital Micrograph V1.81.78.

### Measurement of Evans blue extravasation

In this terminal procedure, anesthesia was induced via isoflurane inhalant (3–4%) and maintained via intramuscular injection of urethane (1.75 x 10^3^ mg/kg). The jugular vein was isolated and cannulated to permit injection of Evans blue dye (320 mg/kg) in lactated Ringer’s solution. After a 30-minute circulating period, thrombin-cleaved recombinant mouse S3ED (rmS3ED) or rmS4ED in lactated Ringer’s solution (200 μl bolus of 50 μg/ml rmS3ED/rmS4ED; generated via 1-hour incubation at 37°C on a rotator) was administered via the jugular vein cannula. For a mouse weighing 25g and estimated blood volume of 1.5 ml, the final concentration of rmS3ED/rmS4ED would be 5.5 μg/ml, whilst that the final concentration of thrombin would be 1.5 U/ml. After 20 minutes, the mouse was exsanguinated and flushed with lactated Ringer’s solution, and the lungs carefully excised. The left upper lobe was frozen for future scanning on an Odyssey CLx infrared imaging system (LI-COR Biotechnology, NE, USA). The right lobes were collected and frozen for future Evans blue quantification analysis, carried out as described previously[15]. Briefly, lobes were homogenized in 1 ml PBS then the dye extracted with 2 ml formamide. The dye was then quantified by constructing a standard curve using freshly made Evans blue in formamide, reading on a spectrophotometer at 620 nm and then extrapolating the unknown values from the standard curve via GraphPad prism.

### Cell culture

Primary human umbilical vein endothelial cells (HUVECs) from pooled donors cultured with endothelial cell basal media and supplementary kit were used up to passage 5. Cells were grown at 37°C and 5% CO_2_.

### Electric cell-substrate impedance sensing (ECIS) assay

HUVECs at passage 4 were seeded at 100% confluency onto gelatin-coated electric cell-substrate impedance sensing (ECIS) arrays (8W10E+) (Applied Biophysics, NY, USA) and used in experiments when cell monolayers were measuring a resistance of approximately 1800–2400 ohms. In experiments testing the ability of untreated SDC ectodomains to induce changes in endothelial monolayer resistance, the arrays were treated with recombinant human (rh) SDC ectodomains, or vehicle (PBS), diluted in endothelial cell basal media. In experiments testing the ability of thrombin-cleaved rhSDC ectodomain fragments to induce changes in monolayer resistance, rhS3ED or rhS4ED (200 μg/ml) (or vehicle; PBS) were incubated with thrombin (50 U/ml) (or vehicle, 0.1% BSA in PBS) in endothelial cell basal media for 1 hour at 37°C on a rotator. The same procedure was followed to test the rhS4ED sourced from E. Coli and thus void of glycosylation. For treatment of ECIS arrays, the final concentration of rhSDC ectodomains was 2 μg/ml, and the final concentration of thrombin was 0.5 U/ml. In some experiments, endothelial cells were pre-treated with Rho kinase inhibitor Y27632 (10 μm) or vehicle (DMSO) for 1 hour before treatment with cleaved rhSDC ectodomains. Alternatively, endothelial cells were pre-treated with PAR1 antagonist RWJ56110 (50 μm) for 1 hour before rhSDC ectodomain treatment.

### Immunofluorescence staining of VE-cadherin and F-actin

HUVECs were grown to 100% confluence on 0.1% gelatin-coated coverslips, then treated with rhSDC ectodomain fragments or vehicle for 2, 4, 6, 8 or 25 minutes before being fixed 2% PFA for 10 minutes then 4% PFA for 10 minutes. HUVECs were washed with 3% BSA/PBS, then permeabilized with 0.1% triton-x in 3%BSA/PBS for 10 minutes. Following further washes, the cells were then blocked with 10% donkey serum in 3%BSA/PBS with 0.01% tween-20 for 60 minutes. HUVECs were then stained with anti-VE-cadherin (1:200, overnight 4°C). The next day, HUVECs were washed and then stained with AlexaFluor 647-conjugated phalloidin (1:250) and AF488-conjugated donkey anti-rabbit (1:500) for 60 minutes at room temperature. Further washes were followed by staining with DAPI hydrochloride (10 μg/ml) for 15 minutes. HUVECs were washed for the final time before the coverslips were mounted on slides with ProLong diamond antifade mountant. Images were taken with a x60 objective using an Olympus FV1200 spectral inverted laser scanning confocal microscope with associated Olympus FluoView software. A total of 6–8 x 1 μm slices were taken and used to create a maximum projection Z-stack.

### Immunofluorescence staining for syndecan ectodomains on HLMVECs following thrombin treatment

Human lung microvascular endothelial cells (HLMVECs) were treated with vehicle or thrombin (10 U/ml) for 1 hour and subsequently fixed. Cells were stained for S1ED (1:200), S2ED (1:100), S3ED (1:100) or S4ED (1:100) for 1 hour at room temperature then washed before secondary antibody (AF488; 1:500; 1 hour room temparature) was applied. Following washes, coverslips were mounted on slides with ProLong diamond antifade mountant. Images were taken using an Olympus FV1200 spectral inverted laser scanning confocal microscope with associated Olympus FluoView software. A total of 6–8 x 1 μm slices were taken and used to create a maximum projection Z-stack.

### Western blot analysis

To visualize thrombin cleavage of SDC ectodomains, rhSDC ectodomains were incubated with thrombin (10 U/ml; 24-hour to correlate with ELISA or 50 U/ml; 1 hour to correlate with ECIS) at 37°C on a rotator. Samples were heated with protein loading buffer at 95°C for 15 minutes, then placed on ice to cool before loading into a 4–20% mini-PROTEAN TGX precast gel (Bio-Rad, CA, USA). Blots were incubated with SimplyBlue SafeStain for 1 hour then washed with 100 ml double-distilled water (ddH_2_O) for 1 hour on a rocker. A second wash in which 30 ml of 20% NaCl was added to the 100 ml ddH_2_O was performed to maximize sensitivity and clarity according to manufacturer instructions. An Odyssey CLx with Image Studio software was used to scan the blots in the 700 nm fluorescence channel.

For recombinant human SDC ectodomains labelled with a C terminal 6-his tag, western blot analysis was performed as described above, but rather than the gels being stained with SimplyBlue, they were instead transferred using Turboblot, blocked with Odyssey blocking buffer PBS and then stained for the 6-his tag to reveal the basic composition of the SDC fragments. Following 5 x 5-minute washes in 0.1% tween in PBS, the blots were stained with secondary antibody donkey anti-mouse 680 (1:500) for 60 minutes at room temperature. After a further 5 x 5-minute washes in 0.1% tween in PBS, the blots were scanned at a wavelength of 700 nm on an Odyssey CLx.

### Statistics and analysis

Graph construction and statistics were carried out using GraphPad Prism V7.0a. All analysis is shown as mean ± S.E.M., with the exception of the third figure where the bar represents the mean value and each circle represents a different donor.

The data in the third, fourth and fifth figures were analyzed with Student’s T-test within each of the four syndecans and their respective treatments. In the seventh figure, the data were analyzed by one-way ANOVA with Tukey multiple comparison testing. In the eighth figure, the error bars on ECIS tracings are standard deviation and represent 3–4 wells within a single experiment. In the eighth and ninth figures, the plot profiles were performed as shown previously [16]. Briefly, a line was drawn across the cell and the intensity along that line measured using ImageJ. The intensity at each position along that line can then be plotted on a graph. Under vehicle conditions, protein expression is primarily at the cell borders, but becomes more dispersed across the cell as the protein expression changes with treatment. Statistical significance was taken as the following: <0.05 (*), <0.01 (**), <0.001 (***), <0.0001 (****), up to 4 significant figures.

## Results

### Endothelial glycocalyx is present in human lung microvasculature

**Fig 1:**
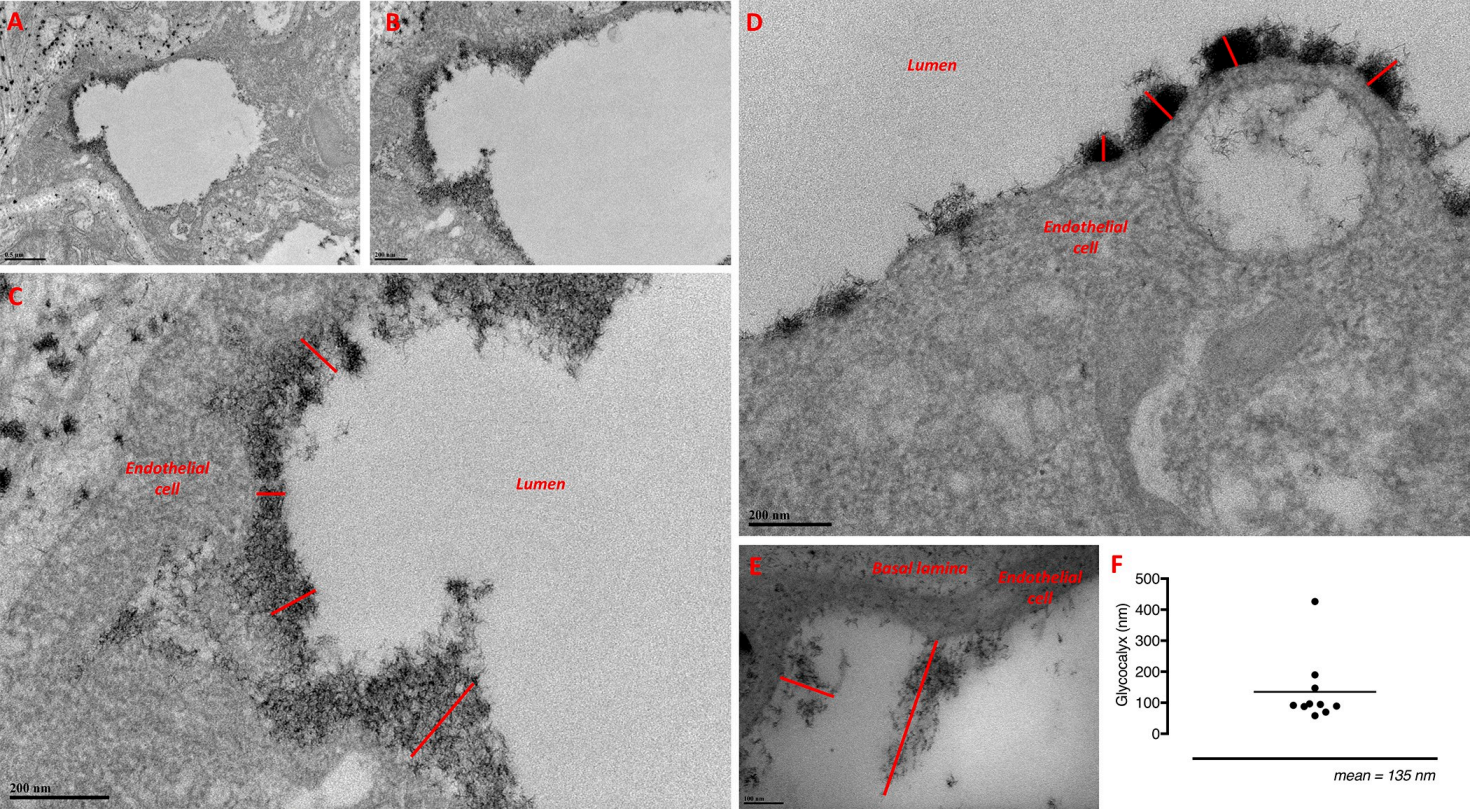
Endothelial glycocalyx structure in human lung. Transmission electron microscopy was used to visualize the endothelial glycocalyx in the microvasculature of human lung tissue. Capillaries demonstrated varying levels of glycocalyx coverage, and the structure of the glycocalyx appeared to differ between vessels. (A, B, C) In this vessel, relatively consistent endothelial glycocalyx coverage across the endothelium can be seen on the left hand side of the image. (D) The endothelial glycocalyx in these vessels appears bush-like and clumped. (E) Glycocalyx that is non-uniform in appearance can be seen attached to endothelial cells and is protruding into the lumen by as much as 400 nm. Scale bars: A 0.5 μm, B, C, D 200 nm, E 100 nm. (F) Analysis of the thickness of the glycocalyx in these vessels suggests an average thickness of 135 nm, but ranging from 58 nm up to 426 nm.

Transmission electron microscopy (TEM) has been used in multiple studies to image the eGCX; the vast majority use animal tissue vasculature, with very few examples in human tissue [17–19]. The eGCX has yet to be demonstrated in the vasculature of the human lung and so TEM was used to ascertain the presence and structure of the eGCX in human lung capillaries. Micrographs confirm the presence of eGCX in human lung capillaries, and also demonstrate the potential heterogeneity of its structure, thickness and coverage. For example, the endothelium in the microvessel in Fig 1C is covered with a continuous layer of eGCX which measures between 100–200 nm thick. In Fig 1D the eGCX is present as ‘bush-like’ clumps (70–95 nm thick) that sit side by side along the luminal surface of the endothelium. The eGCX shown on the endothelium of the vessel in Fig 1E is far sparser and only thinly coats the vasculature, yet the eGCX components appear to protrude further into the lumen by as much as 400 nm.

### All four syndecan family members are expressed in human lung microvasculature

**Fig 2:**
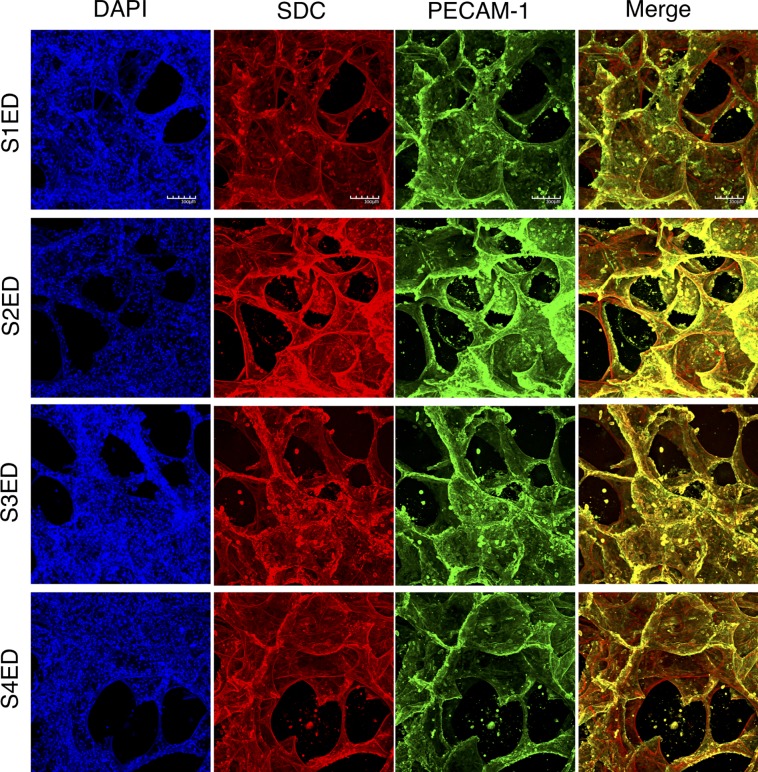
Syndecan family is expressed in microvasculature of human lung. Sections of agarose-inflated human lung were stained for each of the four syndecan ectodomains and the endothelial cell marker, PECAM-1. Confocal images demonstrate co-localization of PECAM-1 with all four syndecan ectodomain proteins, indicating expression throughout the pulmonary microvasculature. Scale bars: 100 μm.

This study was particularly interested in the shedding and signaling properties of the SDC family of proteoglycans which constitute an important component of the eGCX. To determine the expression of S1ED, S2ED, S3ED and S4ED in lung microvasculature, sections (200 μm) of agarose-inflated *ex vivo* human lung were stained for each SDC ectodomain protein, PECAM-1 and cell nuclei. Confocal microscopy revealed the expression of all four SDC members on human lung endothelium, as indicated by the co-localization of endothelial cell marker PECAM-1 with SDC ectodomain proteins (Fig 2). The expression of the SDC ectodomains in human lung can be seen in more detail in movies 1–4.

### Thrombin mediates syndecan-3/-4 cleavage in *ex vivo* human lung slices

**Fig 3:**
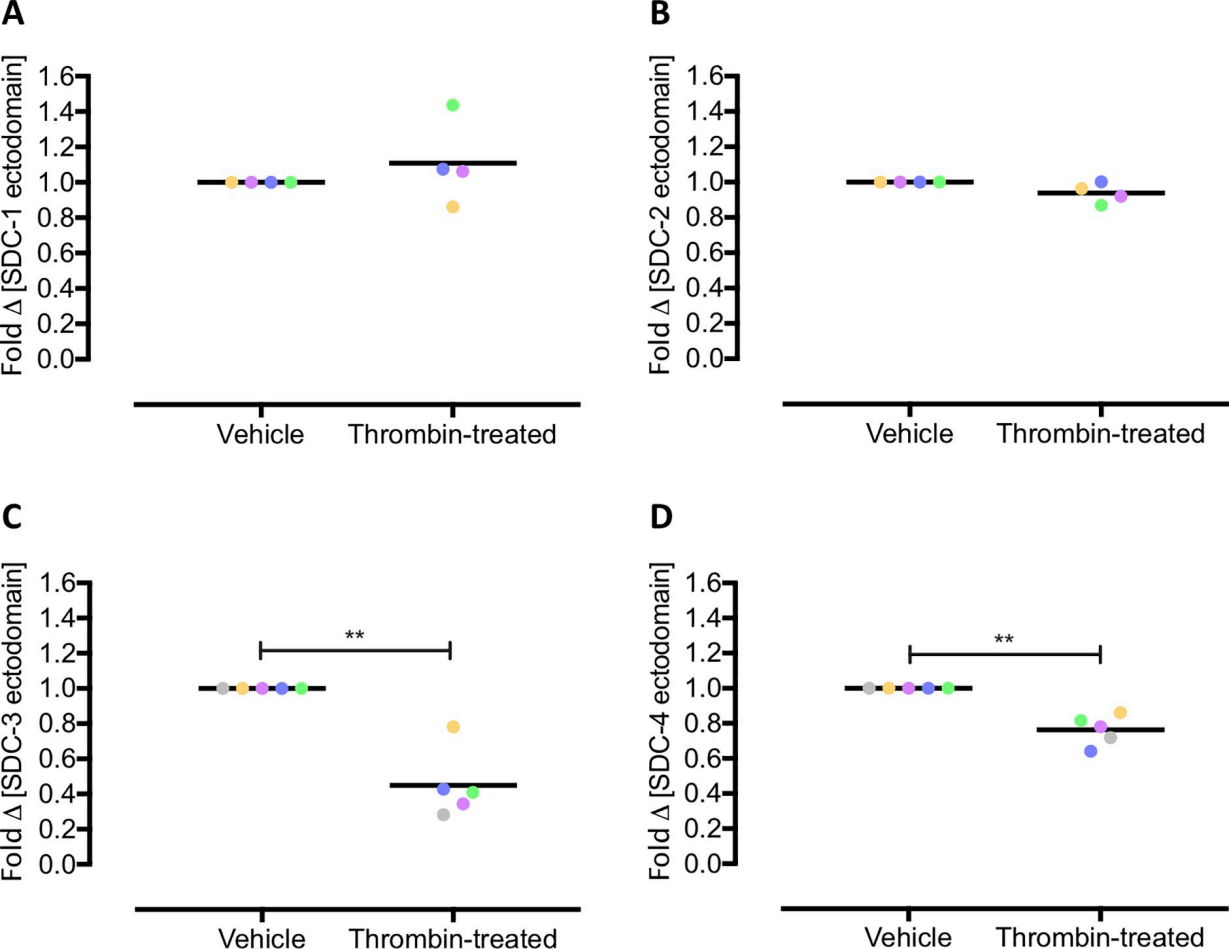
Thrombin-induced changes in syndecan ectodomain proteins from ex vivo human lung. ELISA was performed to measure the concentration of each of the syndecan ectodomains in the supernatant of fresh human lung slices treated with vehicle or thrombin. Thrombin treatment (10 U/ml; 24 hours) of *ex vivo* human lung generated no difference in concentration of human (A) S1ED (n = 4, p = 0.47) or (B) S2ED (n = 4, p = 0.13). However, the concentrations of both (C) S3ED (n = 5, p = 0.0072) and (D) S4ED (n = 5, p = 0.006) were significantly decreased following thrombin treatment.

SDC ectodomains have been reported to contain cleavage sites for a number of different proteases including thrombin, mostly determined through *in vitro* studies co-incubating recombinant protease and SDC ectodomain [5]. Here, we sought to investigate the ability of thrombin, a protease generated in increased amounts during sepsis and thrombotic disease states, to cleave SDC ectodomain in *ex vivo* lung tissues. It is important to note that the use of precision cut lung slices combined with ELISA to determine the concentration of soluble molecule in the supernatant has been done previously [20–22] but it has its drawbacks in that the cellular source of the soluble molecule of interest is unknown. Nevertheless, the method provides a useful indication here of syndecan cleavage using valuable human lung tissue. Treatment of fresh human lung slices with thrombin (10 U/ml, 24 hours) had no effect on S1ED or S2ED concentration within the supernatant as measured via ELISA (Fig 3A and 3B). In contrast, the concentration of S3ED and S4ED was significantly decreased within the supernatant following thrombin treatment (Fig 3C and 3D). This decrease could suggest cleavage at sites throughout the ectodomain, rather than simply at a site juxtaposed to the cell membrane, rendering the S3ED and S4ED undetectable by the ELISA antibodies.

### Thrombin treatment of ex vivo human lungs slices decreases S3ED and S4ED expression

**Fig 4:**
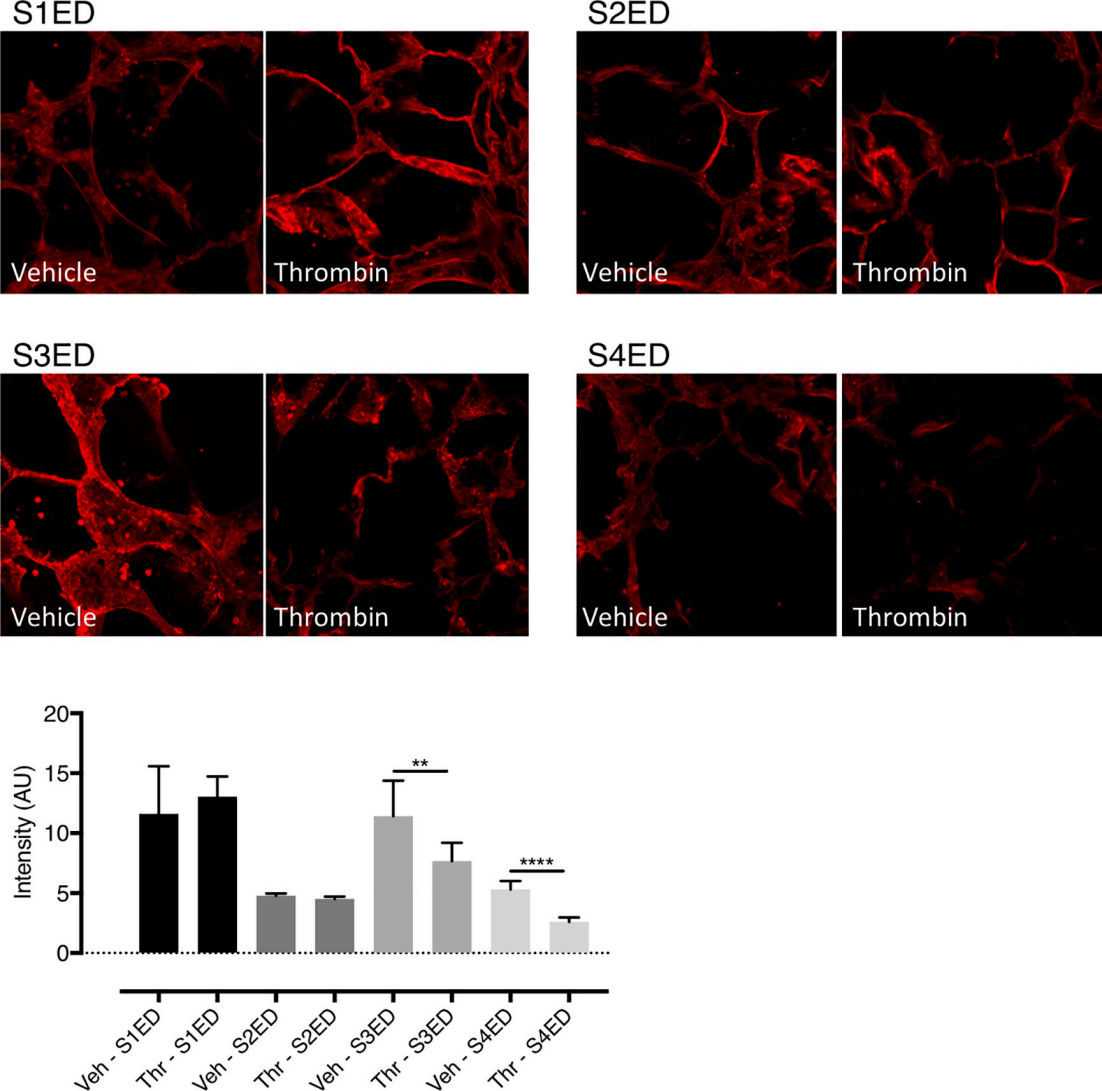
Thrombin cleaves S3ED and S4ED from ex vivo human lung slices. Human lung slices were treated with vehicle or thrombin (24hr; 10 U/ml) and then imaged on a confocal microscope to assess the effect on SDC ectodomain expression. The data shown in Fig 4 suggest that both S3ED (n = 1) and S4ED (n = 1) have decreased expression in human lung slices following thrombin treatment.

To determine if thrombin can affect the expression of SDC ectodomains throughout ex vivo human lung slices, we treated human lung slices with thrombin (24hrs/ 10 U/ml) and imaged with a scanning confocal microscope. The intensity of single confocal slices from the same sample was analyzed using ImageJ and the data displayed in Fig 4. Our analysis suggests that thrombin treatment decreases the expression of both S3ED and S4ED throughout the ex vivo human lung slices.

### All four syndecans are expressed on human lung microvascular endothelial cells and treatment with thrombin decreases S3ED expression

**Fig 5:**
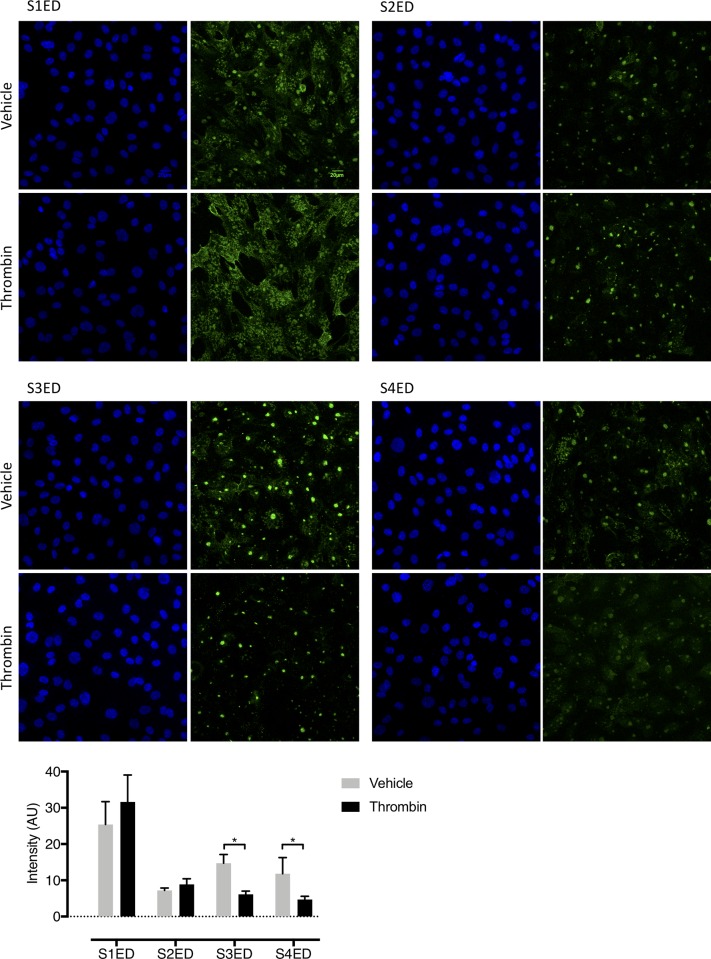
Thrombin cleaves S3ED from human lung microvascular endothelial cells. Immunofluorescence was used to verify expression of syndecan ectodomains on human lung microvascular endothelial cells (HLMVECs). All four syndecans were found expressed on HLMVECs, with thrombin treatment (1 hour) significantly decreasing the expression of S3ED and S4ED (p<0.05).

To confirm expression of syndecan proteins on the surface of human lung microvascular endothelial cells (HLMVECs), we stained for syndecan ectodomains on cells treated with vehicle or thrombin. As shown in Fig 5, HLMVECs express all four syndecan family members. In addition, we found that treatment of HLMVECs with thrombin significantly reduced the expression of both S3ED and S4ED (p<0.05).

### Thrombin generates S3ED and S4ED fragments, and 6-his tag labelling aids probable fragment identity

**Fig 6:**
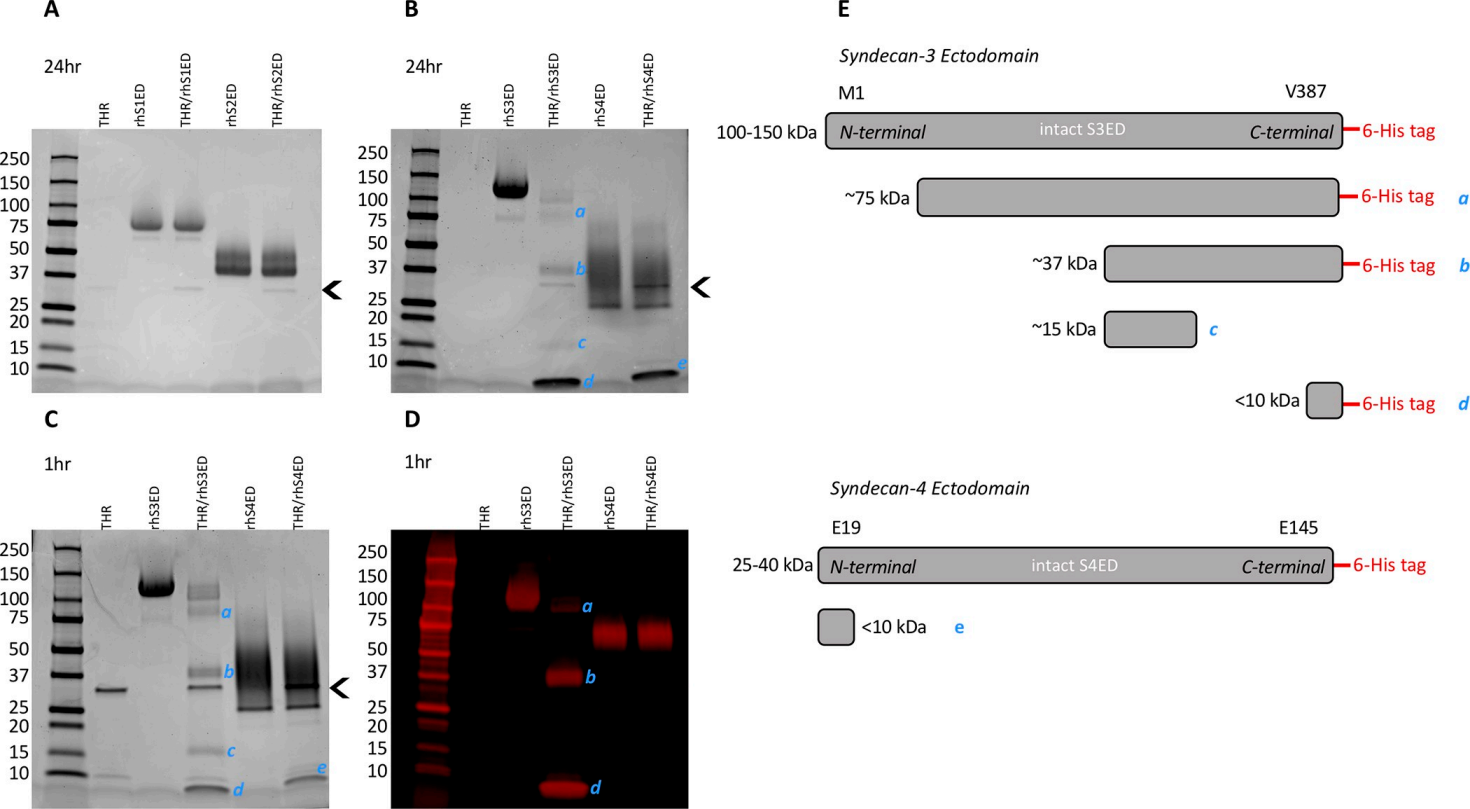
Thrombin generates syndecan ectodomain fragments. To aid our understanding of the decrease in S3ED/S4ED proteins, western blots were carried out with recombinant syndecan ectodomains treated with thrombin. (A) Neither rhS1ED or rhS2ED were found to be cleaved by thrombin (10 U/ml, 24 hours). (B) In contrast, rhS3ED and rhS4ED were both cleaved by thrombin (10 U/ml, 24 hours) to produce 4 and 1 fragments respectively (labelled *a-e*). (C) A shorter treatment time with thrombin of 1 hour was sufficient to cleave and generate the same rhS3ED (*a-d*) and rhS4ED (*e*) fragments. (D) Probing of the SDC fragments with anti-6-his tag showed three of the four rhS3ED fragments all contain the C-terminal 6-his tag, whilst the rhS4ED fragment generate through thrombin treatment did not contain the 6-his tag. (E) A schematic to illustrate the probable identities of some of the different SDC ectodomain fragments generated by thrombin based on evidence from western blots. Western blots shown are representative images of n = 3. Arrowhead indicates position of thrombin on blot.

Since the ELISA data demonstrated a decrease in S3ED and S4ED concentration following thrombin treatment, it was hypothesized that the ectodomains were being cleaved by thrombin into two or more fragments. Western blot analysis supported this, revealing that neither the recombinant human (rh)S1ED (200 μg/ml) nor rhS2ED were cleaved by thrombin (10 U/ml; 24 hours) (Fig 6A). In Fig 6B, there was a clear decrease in rhS3ED, with cleavage by thrombin generating four fragments; one fragment at approximately 75kDa (*a*), a second at approximately 37kDa (*b*), a third at approximately 17kDa (*c*) and a fourth very small fragment at <10kDa (*d*). The rhS4ED band does not appear to decrease in intensity or molecular weight following thrombin treatment; however, a second strong band was produced indicating the production of a very small fragment of <10 kDa (*e*). A shorter (1 hour) length of thrombin-treatment time was tested and found to produce identical S3ED and S4ED fragments (a-e) (Fig 6C). The rhS3ED and rhS4ED are labelled with a C-terminal 6-his tag. When labelled with a 6-his tag antibody, S3ED fragments of 75kDa (*a*), 37kDa (*b*) and <10kDa (*d*) all showed positive staining in the C-terminus, whilst the 17 kDa fragment (*c*) did not. Interestingly, the small S4ED fragment (*e*) seen on the Coomassie blue-stained blots did not label with the 6-his tag antibody, indicating it is likely part of the N-terminal end of the ectodomain protein (Fig 6D). Fig 6E displays a schematic to illustrate the possible identities of the different S3ED and S4ED fragments based on the findings from the Coomassie blue and the 6-his tag blots.

### Syndecan-3/-4 ectodomain fragments decrease endothelial barrier resistance via rho kinase, and even in presence of PAR1 antagonist

**Fig 7:**
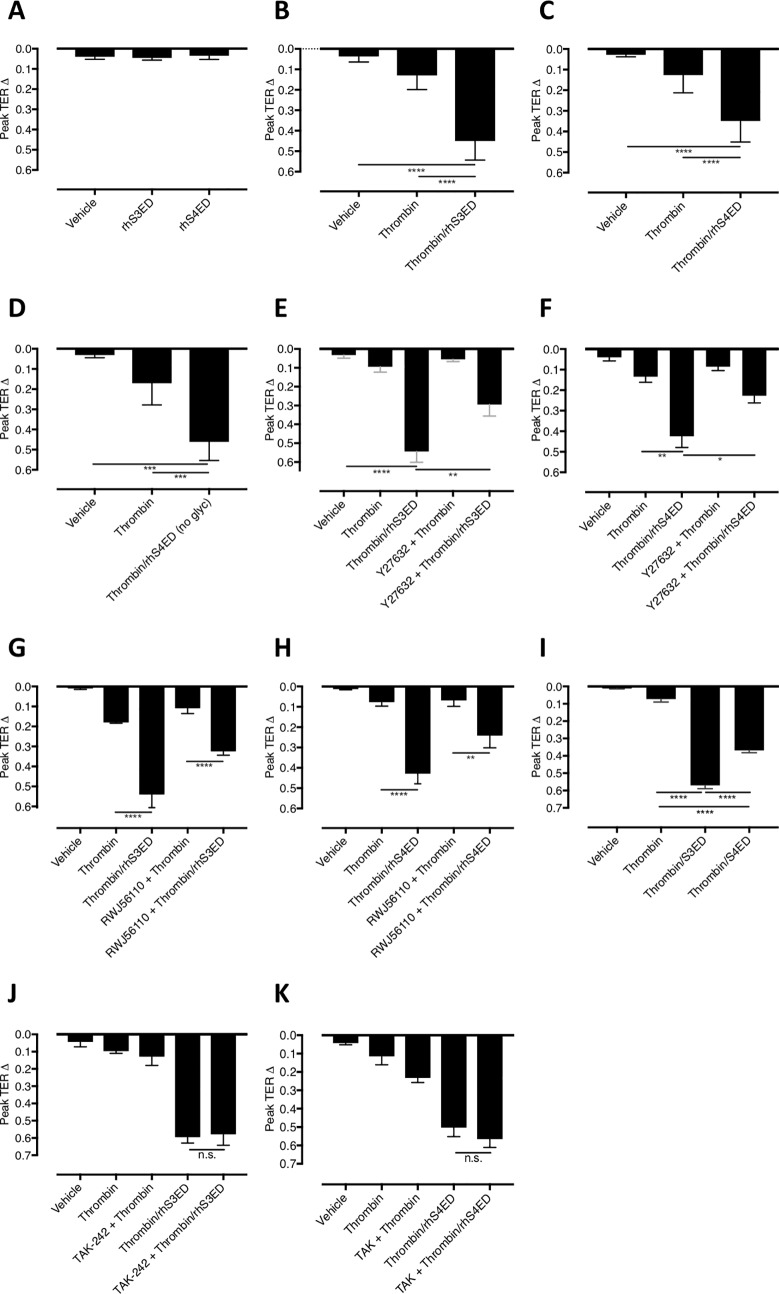
Syndecan ectodomain fragments decrease endothelial resistance via rho kinase. The ability of syndecan ectodomains or their fragments generated following thrombin cleavage to modulate changes in endothelial barrier resistance was assessed using an electric cell-substrate impedance sensing assay, which measures changes in endothelial barrier resistance. (A) Intact rhS3ED (n = 4, p = 0.95) and rhS4ED (n = 4, p = 0.55) had no effect on transendothelial electrical resistance (TER). (B) rhS3ED fragments induced a highly significant drop in TER (n = 6, p<0.0001). (C) rhS4ED fragments significantly decreased TER (n = 8, p = 0.0002). (D) Fragments of non-glycosylated form of rhS4ED also generated significant decreases in TER (n = 5, p = 0.0011). (E) Pretreatment with Y27632 (10 μM, 1hr) significantly inhibits S3ED fragment-induced decreases in peak TER change (n = 4, p = 0.0045). (F) Pretreatment with Y27632 (10 μM, 1hr) significantly inhibits S4ED fragment-induced decreases in peak TER change (n = 4, p = 0.01). (G) Because thrombin is still present in the syndecan ectodomain fragment mixture, albeit at the low concentration of 0.5 U/ml, the ECIS assays were carried out following pre-treatment with the PAR1 receptor antagonist, RWJ56110. In the presence of RWJ56110, rhS3ED fragments mediated a significant decrease in TER response (n = 3, p<0.0001). (H) Following pre-treatment with RWJ56110, rhS4ED fragments induced a significant decrease in TER (n = 3, p<0.01). (I) S3ED (n = 4, p<0.0001) and S4ED (n = 4, p<0.0001) fragments significantly decrease TER response in human lung microvascular endothelial cells. Furthermore, S3ED fragments have a significantly bigger effect on TER than S4ED fragments (n = 4, p<0.0001). (J) TLR4 inhibitor, TAK-242, had no effect of the barrier disrupting effects of the S3ED (p = ns, n = 3) and (K) S4ED fragments (p = ns, n = 3).

With the knowledge that thrombin is able to cleave S3ED and S4ED from *ex vivo* human lung tissue and generate multiple ectodomain fragments, we tested whether or not S3ED and S4ED fragments can modulate endothelial barrier resistance. This was achieved using the electric cell-substrate impedance sensing (ECIS) assay which can record changes in transendothelial electrical resistance (TER) as a measure of endothelial cell-cell adhesive barrier integrity [23]. HUVECs grown on ECIS arrays and subsequently treated with unaltered rhS3ED or rhS4ED produced no change in TER, suggesting that full-length syndecan ectodomains do not directly damage barrier integrity (Fig 7A). For completeness, full length S1ED and S2ED were also tested for their ability to modulate TER but no effect was seen (S1 Fig). In contrast, when rhS3ED and rhS4ED (200 μg/ml; final concentration 2 μg/ml) were pre-treated with thrombin (50 U/ml; final concentration 0.5 U/ml; 1 hour) to produce ectodomain fragments, they caused a significant decrease in TER across the HUVEC monolayer (Fig 7B and 7C). Fig 7D shows that rhS4ED produced using a non-mammalian expression system which renders a core protein void of glycosylation, also produces a significant decrease in TER response, indicating that glycosylation was not required for the syndecan cleavage products to affect the barrier property. To investigate the downstream signaling mechanisms involved in the S3ED and S4ED fragment response, cells on ECIS arrays were pretreated with rho kinase inhibitor, Y27632. The data indicate a significant decrease in the change in peak TER response from both the S3ED (n = 4, p<0.01) and S4ED (n = 4, p<0.05) (Fig 7E and 7F) indicating a role for the rho kinase pathway in the SDC fragment response on endothelial barrier. Although the final concentration of thrombin within the ECIS array is very low (0.5 U/ml), we wanted to exclude any role of thrombin in the SDC ectodomain-induced drops in TER, thus cells were pre-treated with RWJ56110 (50 μM), a thrombin receptor PAR1 antagonist, prior to treatment with rhS3ED/rhS4ED fragments. In the presence of the PAR1 antagonist, rhS3ED and rhS4ED fragments both continued to produce highly significant decreases in TER (Fig 7G and 7H), suggesting the residual amount of thrombin in the treatment had a minimal effect in the TER response to syndecan ectodomain fragments. The effect of S3ED and S4ED fragments was also assessed in human lung microvascular endothelial cells, with analysis suggesting a similar decrease in TER, particularly by the S3ED fragments (Fig 7I). Additional ECIS experiments indicate a lack of involvement of the TLR4, since an inhibitor for this receptor did not affect the S3ED or S4ED fragment-induced TER drop (Fig 7J and 7K).

### Syndecan ectodomain fragments cause VE-cadherin disorganization and F-actin stress fiber formation, effects partially rescued by the Rho kinase inhibitor Y27632

**Fig 8:**
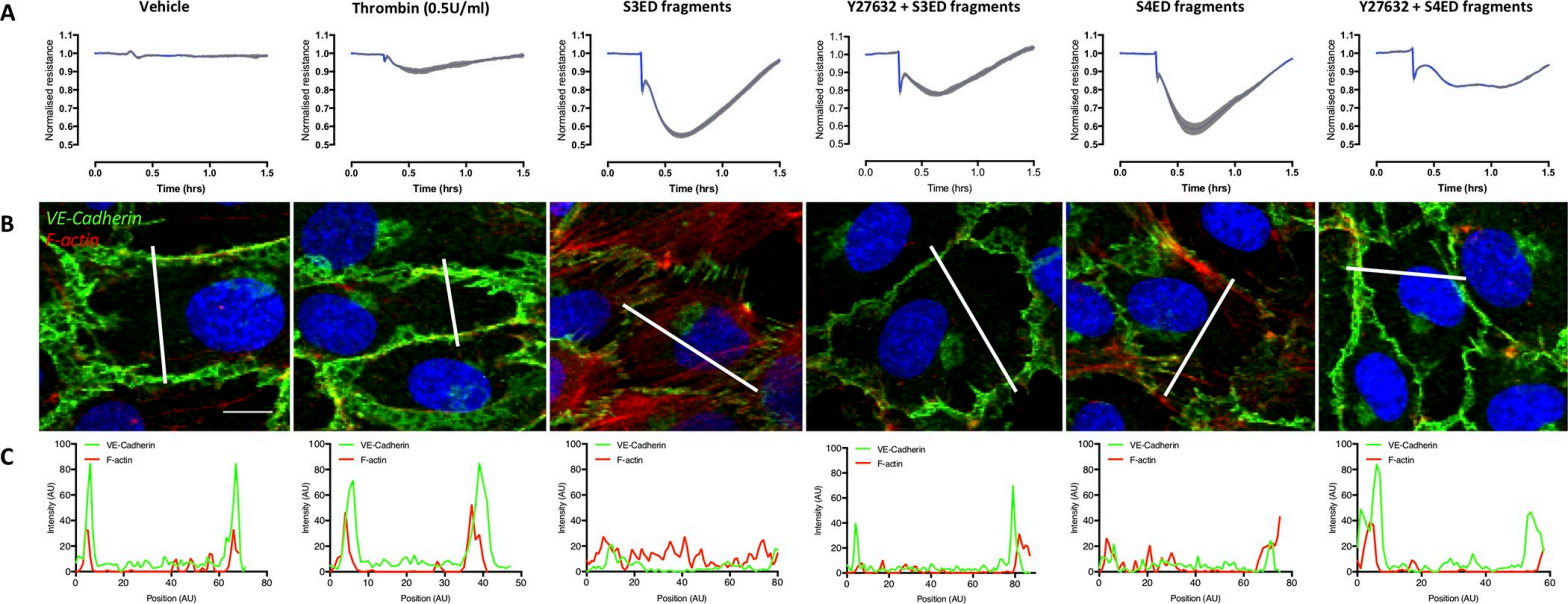
Syndecan ectodomain fragments cause VE-cadherin disorganization and F-actin stress fiber formation. (A) Representative ECIS traces show Rho kinase inhibitor (Y27632, 10 μM, 1hr pre-treatment) blocks the TER changes induced by rhS3ED and rhS4ED fragments. (B) Immunocytochemistry indicates that, compared to thrombin (0.5 U/ml) alone, rhS3ED fragments and rhS4ED fragments (25 minutes treatment) both mediated significant VE-cadherin disorganization and F-actin stress fiber formation, effects that are inhibited in the presence of Y27632 (10 μM, 1hr pre-treatment). (C) Plot profile analysis of the VE-cadherin and F-actin staining shows that, under vehicle or low concentration thrombin, expression of these two proteins peaks at the intercellular junctions where the barrier remains intact. Following rhS3ED or rhS4ED fragment treatment, the plot profile demonstrates clear VE-cadherin disorganization and strong F-actin fiber stress formation throughout the cells. Furthermore, this analysis highlights the ability of rho kinase inhibitor, Y27632, to prevent the junction disorganization. Scale bar = 10 μm.

To investigate the molecular events in the endothelial structure that lead to impaired barrier integrity caused by the S3ED and S4ED fragments, we carried out immunocytochemistry to characterize adherens junction discontinuity and F-actin stress fiber formation, molecular responses known to cause paracellular hyperpermeability. The representative ECIS traces shows a rapid drop in transendothelial electrical resistance immediately after treatment with S3ED and S4ED fragments (Fig 8A). This drop in TER was diminished in cells pre-treated with Rho kinase inhibitor, Y27632 (Figs 7E, 7F and 8A). Under control conditions Vascular Endothelial (VE)-cadherin, an adherens junction protein, is located at the cell-to-cell contacts. There is clear disorganization of VE-cadherin and increased F-actin stress fiber formation following treatment with the S3ED and S4ED fragments, which was prevented by the Rho kinase inhibitor, Y27632 (Fig 8). The images are supported by plot profile analysis of the protein expression as shown in Fig 8C. Together, these data suggest a role for the Rho pathway in mediating the TER effects of the SDC ectodomain fragments, and that both cytoskeleton and junction organization is altered.

### VE-cadherin disorganization and F-actin stress fiber formation occurs rapidly

**Fig 9:**
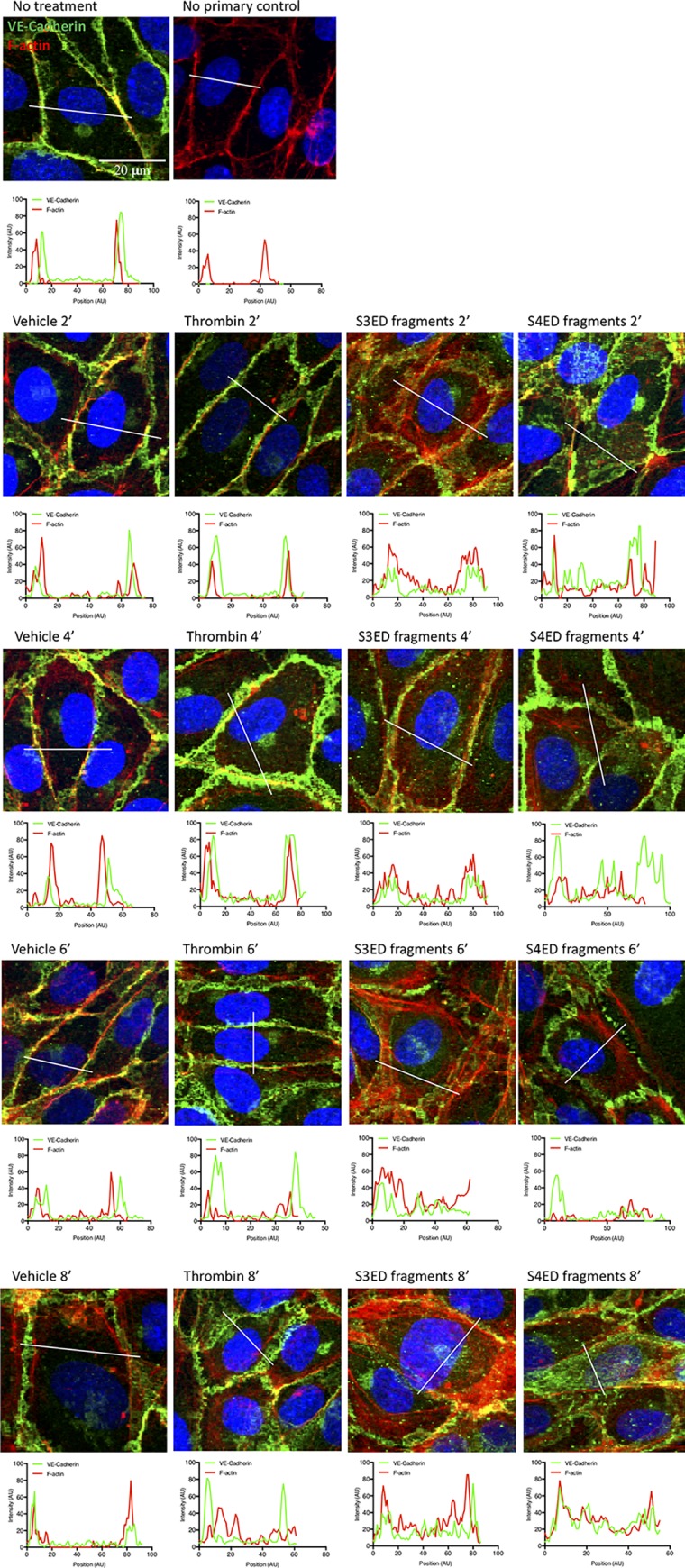
Rapid VE-cadherin disorganization and F-actin stress fiber formation in response to S3ED and S4ED fragments. HUVECs were treated with S3ED and S4ED fragments for 2, 4, 6 and 8 minutes, then stained for VE-cadherin and F-actin. The images show a clear disorganization of VE-cadherin away from the cell-cell junctions and strong F-actin stress fiber formation at these early time points. This observation is supported by the plot profile analysis demonstrating loss of VE-cadherin at the adherens junctions and increased F-actin expression throughout the cells following fragment treatment.

The response to S3ED and S4ED fragments is rapid (Fig 9A). To understand the mechanism behind this response, we stained HUVECs for VE-cadherin and F-actin at the early time points of 2, 4, 6 and 8 minutes. As shown in Fig 9, cells displayed VE-cadherin disorganization with decrease expression at the cell-cell junctions in response to both rhS3ED and rhS4ED fragments. Furthermore, strong F-actin stress fiber formation was seen following treatment with the syndecan fragments.

### Syndecan-3 ectodomain fragments increase plasma protein extravasation in mouse lung

**Fig 10:**
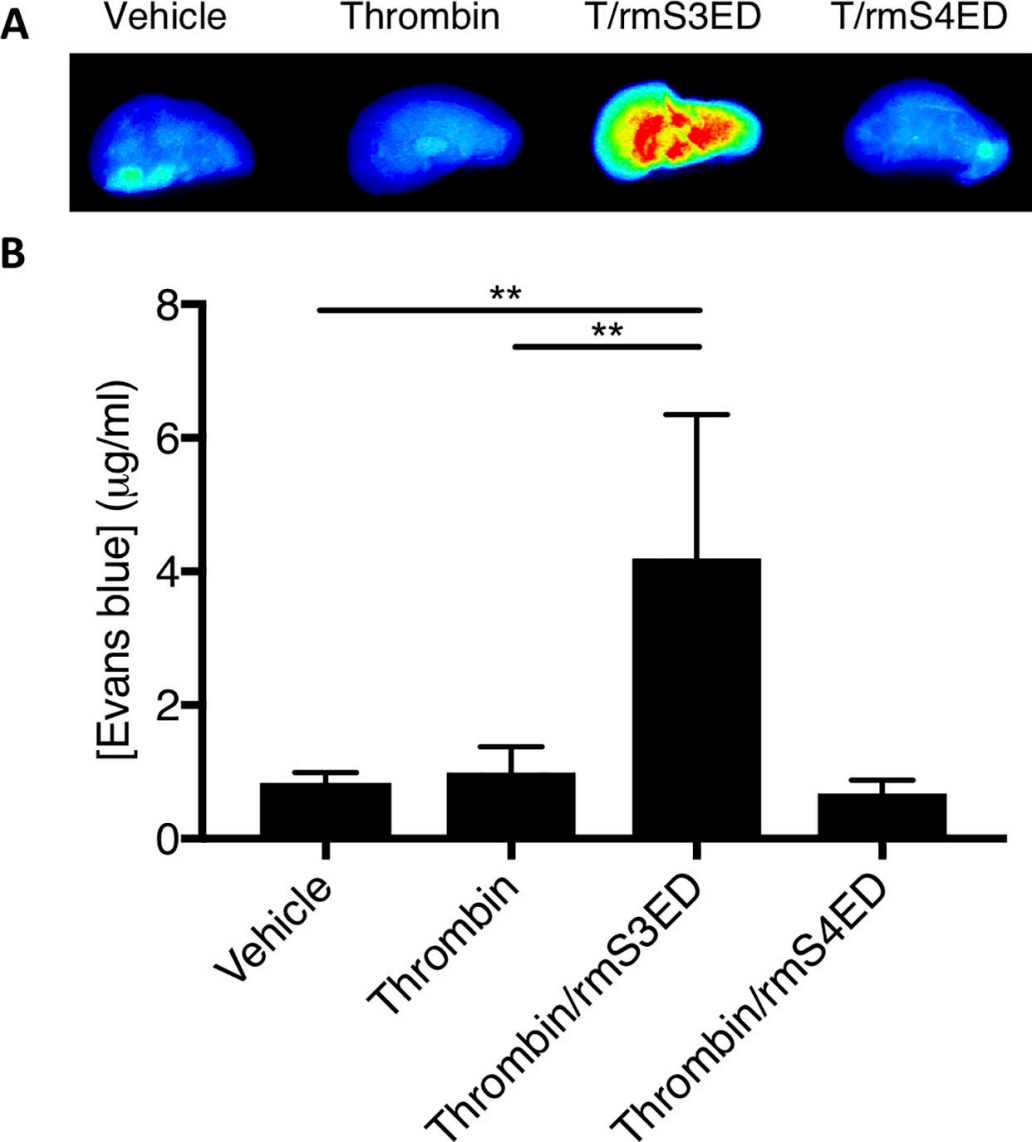
Syndecan-3 ectodomain increases vascular leakage in mouse lung. Evans blue dye binds with high affinity to serum albumin, thus its leakage from the vasculature into the surrounding tissue when an insult is received can be a useful indicator of disrupted endothelial barrier integrity. (A) When administered intravenously, rmS3ED fragments (5.5 μg/ml, 20 minutes, n = 4) increased Evans blue dye extravasation within the lungs of mice compared to controls, as evidenced by the increase in dye presence shown by the heat map. Interestingly, the rmS4ED fragments did not show any increase in plasma leakage within the pulmonary vasculature (5.5 μg/ml, 20 minutes, n = 4). (B) Quantitative analysis of Evans blue dye within mice lungs confirms visual evidence that rmS3ED fragments significantly (p<0.01) increase Evans blue dye extravasation, and therefore plasma leakage, into the surrounding tissue. The lack of effect of rmS4ED fragments is also confirmed by the quantitative analysis.

Having demonstrated thrombin-induced cleavage of S3ED and S4ED in human lung *ex vivo*, and the production of S3ED/S4ED fragments capable of decreasing endothelial barrier resistance *in vitro*, our study next assessed the ability of S3ED/S4ED fragments to mediate endothelial barrier changes *in vivo*. Evans blue is a dye that binds with a high affinity to plasma albumin, and thus its extravasation from the circulation into the tissue provides a useful first approximation of barrier loss *in vivo*. Compared to vehicle controls, mice administered recombinant mouse (rm)S3ED fragments exhibited increased extravasation of Evans blue in their lungs, as evidenced by the scan and pseudo-heat map of representative left lobes (Fig 10A) and the quantification of the right lobes (Fig 10B). However, when treated with rmS4ED fragments, no increase extravasation of Evans blue was observed (Fig 10A and 10B).

## Discussion

Syndecans are key components of the endothelial glycocalyx (eGCX). Multiple studies have reported increased levels of SDC ectodomains in the plasma of trauma and sepsis patients [6,7,9,10], evidence that the eGCX is subject to degradation and injury under such conditions. The loss of this protective layer results in an impaired barrier where inflammatory agonists and cells can more easily access the endothelium [1,13]. In this study, we considered the possibility that the injury and cleavage of eGCX components results in vascular permeability consequences beyond the well-established loss of barrier effect [24]. We propose that proteases such as thrombin can cleave eGCX components to produce soluble hyperpermeability molecules capable of acting as signaling mediators that promote endothelial dysfunction. A similar concept was recently proposed for heparan sulfate fragments released during eCGX injury [25].

Whether endothelial surface glycocalyx exists across the microvasculature of different organs and tissues has been a subject of controversy. We performed transmission electron microscopy (TEM) to characterize the eGCX within the vasculature of human lungs, which has not previously been studied. Electron micrographs revealed clear evidence for the presence of the eGCX in human lung capillaries, and also demonstrated the heterogeneity of its structure. The bush-like eGCX structures, such as those seen in some of the micrographs in this study, are similar to those published in the vasculature of rodent vessels [26,27]. Clearly, it is difficult to know what effect fixation has on the structure and appearance of the eGCX. In 2014, Yang *et al*. generated impressive *ex vivo* images of the eGCX ultrastructure in human aqueous humor outflow pathways, produced by similar Alcian blue staining methods to those used in the current study [18]. The authors showed spikey hair-like eGCX structures, which were non-uniform, particularly between the different vessels within the eye. TEM was used previously to visualize the eGCX in the human umbilical vein [17]. In a third study, the glomerular capillaries from human donor kidneys were stained and then imaged by TEM [19]. Although not the primary purpose of their study, the authors did note the presence of some eGCX, albeit a very sparse and thin layer, particularly in comparison to some of the images from both our study and the aforementioned Yang *et al*. The eGCX has been shown in the pulmonary capillaries of mouse lungs using TEM and scanning electron microscopy, with the authors also demonstrating disruption of the eGCX structure following LPS-induced septic lung injury [28]. Importantly, we show the first evidence of the presence of the eGCX and its ultrastructure on human lung endothelium. Together, these data highlight the heterogeneity in eGCX between the different tissues, vascular beds and even within the same vessel type.

For this study we were particularly interested in the syndecan (SDC) proteins, which constitute key components of the eGCX. SDC ectodomains protrude from the endothelial cell surface into the lumen of the vessel. Here, we used precision cut lung slices to demonstrate positive staining for all syndecan ectodomains throughout the human lung vasculature; they colocalized with endothelial cell marker, PECAM-1, confirming their expression on the endothelial cell surface. A previous study investigated the expression of each of the SDC proteins in HUVECs, using qRT-PCR to demonstrate an abundance of SDC-3 and SDC-4, but low expression of SDC-1 and SDC-2 [29]. Chui *et al*. found expression of all four SDC family members within human placentas, with each SDC differentially expressed amongst the various cell types [30]. A study investigating the relationship between atherosclerotic plaque burden and SDC-4 expression found that the endothelial cells of SDC-4 knockout mice are poorly aligned and that these mice are susceptible to the formation of plaques in normally resistant areas [31]. Although there is evidence of increased circulating levels of SDC ectodomains following lung injury [9], to the best of our knowledge the expression of SDC proteins in animal or human pulmonary vasculature has not been studied. The imaging data in the current study provide novel evidence for the presence and characteristics of the eGCX in human lung microvasculature, and in particular, that SDC proteins contribute to the structure of the pulmonary endothelial barrier.

A number of sheddases expressed on cell membranes, including MMPs and ADAM (a disintegrin and metalloprotease) metalloendopeptidases, are believed to cleave transmembrane molecules such as SDC and release their ectodomains [3,4]. Other proteases in the circulation, including secreted MMPs, plasmin and thrombin, have been shown to be able to further degrade the ectodomains into fragments [2,3,5]. In our ELISA analysis of supernatant from fresh human lung slices treated with thrombin, we did not observe changes in the concentration of S1ED or S2ED, yet the concentrations of full-length S3ED and S4ED were significantly decreased. It is important to note that the supernatant collected from the *ex vivo* human lung slices, similar to work published previously [20–22], would contain the secreted molecules of all the different cell types in the tissue and so the change in S3ED and S4ED cannot necessarily be attributed solely to endothelial cells.

To complement these data, we also performed confocal microscopy to look at expression of SDC ectodomains in human lung slices treated with thrombin and found decreased expression of both S3ED and S4ED. The limitation of these data is that, like the ELISA findings, the results account for SDC ectodomain expression throughout the lung not simply endothelial cell expression alone. To this end, we also carried out immunofluorescence experiments to image and analyze expression of each of the SDC ectodomains on human lung microvascular endothelial cells (HLMVECs) following treatment with thrombin, finding decreased expression of S3ED and S4ED on these endothelial cells.

Additionally, we carried out western blot analysis and, consistent with the aforementioned data, confirmed that thrombin does indeed cleaves intact rhS3ED into four fragments. Thrombin treatment also produced a rhS4ED fragment of <10 kDa, although no noticeable decrease in the level of intact rhS4ED was seen. It is plausible that the cleavage of such a small fragment of rhS4ED would not cause visible change in the molecular weight of the protein and consequently its appearance on the blot. Intriguingly, prior research identified a thrombin cleavage site within the S4ED at R36 [3], located at the N-terminal end of the ectodomain. The rhS4ED used in the current study is constructed of amino acids 19–145. With a possible cleavage site at R36, E19 to R36 would render an 18-residue protein fragment of 2.1 kDa (determined using protein molecular weight calculator accessed at www.bioinformatics.org/sms). Of note, this region would be void of any known glycosylation branches. We find that this fragment lacks the 6-his tag, and thus is indeed likely from the N-terminal region of the rhS4ED. This has been illustrated in the schematic shown in Fig 4E, alongside the possible identities of the S3ED fragments. These data are the first to show thrombin cleavage of rhS3ED to produce fragments of the core ectodomain protein and support previous evidence for a thrombin cleavage site in the N-terminal region of the S4ED. Given the presence of both S3ED and S4ED in human pulmonary vasculature, the ability of thrombin to cleave these proteins and generate smaller fragments is of interest. In future studies, it would be interesting to carry out protein sequencing to identify the precise sequence of the different fragments and to conduct further experiments to determine which fragment(s) are responsible for inducing decreases in TER.

The second part of this study was focused on understanding the ability of SDC ectodomain proteins to act as soluble hyperpermeability signaling molecules, in particular once cleaved by the protease thrombin into ectodomain fragments. Previous studies have shown that the S2ED can act via the CD148 receptor [32] to mediate effects on angiogenesis [33,34] and angiogenic sprouting [35], as well as fibroblast migration, contraction and proliferation [36]. Similarly, both the S3ED and S4ED have been implicated in angiogenesis [37,38], whilst the S4ED also has proposed roles in endothelial cell alignment [31] and wound healing [29]. However, until now, the role of SDC ectodomains in endothelial barrier dysfunction was unknown. We assessed the ability of rhS3ED and rhS4ED to mediate changes in endothelial barrier resistance. Whilst intact rhS3ED and rhS4ED did not mediate any change in TER response, when first pre-treated with thrombin to generate ectodomain fragments, both rhS3ED and rhS4ED mediated significant decreases in endothelial barrier resistance. This change in TER was concentration-dependent, with 2000 ng/ml of either S3ED or S4ED fragments inducing the highest decrease in barrier resistance (S3 Fig).

In order to rule out the likelihood that the effects were due to the glycosaminoglycans covalently bound to the core protein, we tested a non-glycosylated rhS4ED, which was synthesized through a non-mammalian expression system, in the same ECIS assay. Importantly, once pre-treated with thrombin, the non-glycosylated rhS4ED fragments decreased resistance in a manner comparable to the regular mammalian NS0-derived rhS4ED, suggesting a lack of involvement of the glycosaminoglycan chains. This supports previous work that suggests the SDC core proteins can signal independently of the glycosaminoglycan chains [39]. Intensive care unit patients with sepsis, trauma or ARDS have increased circulating levels of SDC-1 [7,9,10,40,41] and SDC-3 [10]. Furthermore, increased levels of SDC-1 were found to be associated with the development of DIC in sepsis patients [42]. Thus our findings that thrombin-cleaved S3ED and S4ED fragments can mediate decreases in transendothelial resistance could be important in such diseases, since circulating levels of thrombin and SDC ectodomains are simultaneously raised. Of note, we carried out separate experiments to determine whether MMP-treated SDC ectodomains could induce similar changes in TER, but saw no effect (S4 Fig). Further permutations of MMPs and SDC ectodomains, at various concentrations and over a range of incubation periods would be important in order to confidently show an absence of any effect of MMP-SDC fragments. Indeed, there are also many other proteases to investigate such as plasmin and ADAMs.

The concept that shortened sequences or fragments of SDC ectodomains can produce more potent effects than the full length SDC ectodomain protein was implicated previously by McFall and Rapraeger in 1997. A truncated form of mouse S4ED consisting of amino acids 56–109 competed more strongly in a cell adhesion assay than a larger form consisting of amino acids 1–120, whilst a peptide formed of amino acids 1–125 failed to compete at all[39]. Of interest, it has recently been demonstrated that ADAM17 can cleave and generate a cytoplasmic C-terminal fragment of the SDC-1 transmembrane domain via intramembrane proteolysis, and that this SDC-1 fragment possesses biological functions including decreasing lung tumor cell metastasis *in vivo* [43]. Our study is the first to demonstrate SDC ectodomain fragments effects on the endothelial barrier. It is noteworthy to mention that *in vitro* thrombin itself can induce significant decreases in TER, but only at higher concentrations than that used here. Our study specifically wanted to determine the effect of SDC ectodomains on endothelial barrier resistance so a minimal concentration of thrombin was critical. Whilst in this study thrombin was used at 0.5 U/ml, when 10 U/ml is applied to HUVECs on an ECIS array, significant reductions in TER are consistently seen [15]. To verify that the TER effects of the SDC ectodomain fragments were not mediated by thrombin and its receptor PAR-1, ECIS experiments were carried out on endothelial monolayers pre-treated with a PAR1 antagonist (RWJ56110)–the primary receptor responsible for thrombin’s barrier-reducing effect *in vitro*. In the presence of RWJ56110, both rhS3ED fragments and rhS4ED fragments continued to produce significant decreases in TER. These results, together with comparison to thrombin alone, allow us to conclude that the TER drop induced by rhSDC ectodomain fragments is separate from those of thrombin directly.

Most of what is known about SDC signaling concerns that which is achieved through the covalently attached glycosaminoglycans [25]. The ability of the core protein of a SDC ectodomain to function as a signaling molecule irrespective of glycosylation was investigated in follow-up research by McFall and Rapraeger, who identified a cell binding domain within the S4ED and demonstrated it’s ability to act as a cell binding substrate and compete fully with full length S4ED [44]. Importantly, neither heparan sulfate nor chondroitin sulfate were able to compete with the S4ED fragment for cell binding. Whiteford *et al*. then showed that the core protein of both S2ED and the S4ED mediated endothelial cell attachment in a β1-integrin-dependent manner [45]. The authors went on to demonstrate that S2ED, specifically the region between amino acids 124–141, can act as a ligand at CD148 [32], and more recently, that the S2ED can inhibit angiogenesis in a CD148-dependent manner [33]. Similar roles for the S1ED [46] and S3ED [37] in inhibiting angiogenesis have been shown. Furthermore, bacterial recombinant S2ED, void of glycosylation, was applied to mouse brain microvascular endothelial cells and shown to induce cell migration and capillary tube formation [47]. These findings highlight the importance of SDC core proteins in endothelial cell physiological and pathological signaling. However, data on the ability of SDC ectodomains to modulate endothelial intercellular junctions is lacking.

The endothelial barrier is maintained by intercellular junctions, the organization of which can be regulated by a number of second messenger pathways. VE-cadherin is an endothelial cell specific molecule and key component of adherens junctions [48]. Here, we found VE-cadherin discontinuity following treatment with rhS3ED or rhS4ED fragments, which is indicative of adherens junction disorganization, a major pathway leading to paracellular leakage. During inflammatory stimulation, junction disorganization often occurs concomitantly with cytoskeleton contraction or actin stress fiber formation; the latter involves RhoA-ROCK signaling [49,50]. Consistently, our study shows that inhibition of the RhoA pathway with Y27632 is able to significantly reduce the SDC fragment-induced decrease in endothelial barrier dysfunction. Both rhS3ED and rhS4ED promoted F-actin stress fiber formation in HUVECs and this was prevented with Y27632. S2ED and S4ED mediated cell adhesion has previously been shown to require the Rho kinase pathway, since Y27632 prevented fibroblast adhesion to SDC ectodomain substrate [51]. The authors found decreased F-actin microfilament bundles as well as reduced focal adhesions. Our data support these findings and provide the novel evidence that the S3ED can also signal via this pathway. Of note, pretreatment of HUVECs on ECIS arrays with myosin light chain kinase inhibitor 18 did not affect SDC fragment-induced TER changes, suggesting a lack of involvement of this pathway (S2 Fig). Since use of Y27632 does not completely block SDC ectodomain-fragment-induced TER changes, future experiments to explore other pathways and signaling mediators such as protein kinase C would be interesting.

The data thus far suggest that S3ED and S4ED are cleaved by thrombin into fragments that possess the ability to act as potent signaling enhancers of endothelial barrier dysfunction. This raises the question, therefore, are these ectodomain fragments functioning via a cell surface receptor, and if so which one. Given that little is known regarding the identity of syndecan receptors, we hypothesized the idea that the fragments may be acting as damage-associated molecular patterns (DAMPs) and as such, used a toll-like receptor 4 (TLR4) inhibitor to test this hypothesis on ECIS. However, the TER response of both rhS3ED and rhS4ED fragments were unaffected by the TLR4 inhibitor, suggesting the involvement of alternative receptor(s) (Fig 7J and 7K). Further studies are warranted to identify the receptor for S3ED and S4ED fragments responsible for the barrier-disrupting effects.

Finally, our study investigated the ability of SDC ectodomains to function as permeability mediators *in vivo*. As indicated in the Evans blue assay, rmS3ED significantly increased extravasation of plasma from the pulmonary vasculature into the surrounding tissue. Interestingly, rmS4ED did not appear to increase plasma leakage within the mice lungs, which is somewhat surprising given its ability to decrease endothelial barrier resistance in the ECIS experiments. The discrepancy might result from the different species or experimental models employed in this study. Results from *in vitro* experiments do not always yield the same results *in vivo*, especially when there is also a species difference to account for.

In summary, we have demonstrated the presence of endothelial glycocalyx, in particular the presence of syndecan ectodomain core proteins, at the endothelial luminal barrier in viable human lungs. We also showed that syndecan ectodomains can be cleaved by thrombin to produce fragments. Mechanistically, we found that rhS3ED and rhS4ED fragments induce significant decreases in endothelial barrier resistance and that this involves the Rho kinase pathway-mediated F-actin stress fiber formation and VE-cadherin junction disorganization. This is the first report regarding the ability of the syndecan family of proteoglycans to act as signaling mediators at the surface of endothelial cells to induce changes in barrier property. Given their expression at the endothelial glycocalyx layer, these findings are of interest in diseases that exhibit increased protease activity and a compromised barrier such as sepsis, ARDS and thrombosis.

## Supporting information

S1 Fig Full length, intact S1ED and S2ED do not mediate significant changes in peak transendothelial electrical resistance (TER). (DOCX)

S2 Fig Myosin light chain kinase inhibitor 18 does not affect the transendothelial electrical resistance (TER) response to S3ED or S4ED fragments in HUVECs. HUVECs at passage 4 were seeded at 100% confluency onto gelatincoated electric cell-substrate impedance sensing (ECIS) arrays (8W10E+) (Applied Biophysics, NY, USA) and used in experiments when cell monolayers were measuring a resistance of approximately 1800–2400 ohms. HUVECs were pre-treated with MLCK inhibitor peptide 18 (10 μm) or vehicle (ethanol) for 1 hour before treatment with cleaved rhSDC ectodomains.
(DOCX)

S3 Fig Concentration-response of S3ED and S4ED fragments on transendothelial electrical resistance (TER) in HUVECs. HUVECs at passage 4 were seeded at 100% confluency onto gelatin-coated electric cell-substrate impedance sensing (ECIS) arrays (8W10E+) (Applied Biophysics, NY, USA) and used in experiments when cell monolayers were measuring a resistance of approximately 1800–2400 ohms. HUVECs were treated with S3ED or S4ED fragments at 100, 300, 1000 or 2000 ng/ml and their TER response measured.
(DOCX)

S4 Fig MMP (2, 9, 14) treated syndecan-3 and syndecan-4 ectodomains do not affect transendothelial electrical resistance (TER) in HUVECs. HUVECs at passage 4 were seeded at 100% confluency onto gelatin-coated electric cell-substrate impedance sensing (ECIS) arrays (8W10E+) (Applied Biophysics, NY, USA) and used in experiments when cell monolayers were measuring a resistance of approximately 1800–2400 ohms. S3ED or S4ED (100 μg/ml) were incubated with MMP2, MMP9 or MMP14 (5 μg/ml) for 2hr at 37°C. These mixtures were then used to treat the HUVECs on the ECIS arrays with final concentrations of 1 μg/ml SDC ectodomain and 50 ng/ml MMP, and the subsequent TER response was recorded and analyzed.
(DOCX)

S1 Table List of reagents used in study, including supplier name and catalog number.
(DOCX)

S1 Movie Syndecan-1 ectodomain expression in human lung. S1ED (red) CD31 (green).
(MP4)

S2 Movie Syndecan-2 ectodomain expression in human lung. S2ED (red) CD31 (green).
(MP4)

S3 Movie Syndecan-3 ectodomain expression in human lung. S3ED (red) CD31 (green).
(MP4)

S4 Movie Syndecan-4 ectodomain expression in human lung. S4ED (red) CD31 (green).
(MP4)
