# Supplementary material for: Thrombin-cleaved syndecan-3/-4 ectodomain fragments mediate endothelial barrier dysfunction
Source: PLoS One. 2019 May 15;14(5):e0214737. doi: 10.1371/journal.pone.0214737 (PMC6519803; doi:10.1371/journal.pone.0214737)
Supplement: S1 Fig — (DOCX) [file pone.0214737.s001.docx]

**S1 Fig:**

Full length, intact S1ED and S2ED do not mediate significant changes in peak transendothelial electrical resistance (TER).
