# Supplementary material for: Thrombin-cleaved syndecan-3/-4 ectodomain fragments mediate endothelial barrier dysfunction
Source: PLoS One. 2019 May 15;14(5):e0214737. doi: 10.1371/journal.pone.0214737 (PMC6519803; doi:10.1371/journal.pone.0214737)
Supplement: S2 Fig — HUVECs at passage 4 were seeded at 100% confluency onto gelatincoated electric cell-substrate impedance sensing (ECIS) arrays (8W10E+) (Applied Biophysics, NY, USA) and used in experiments when cell monolayers were measuring a resistance of approximately 1800–2400 ohms. HUVECs were pre-treated with MLCK inhibitor peptide 18 (10 μm) or vehicle (ethanol) for 1 hour before treatment with cleaved rhSDC ectodomains. (DOCX) [file pone.0214737.s002.docx]

**S2 Fig:**

Myosin light chain kinase inhibitor 18 does not affect the transendothelial electrical resistance (TER) response to S3ED or S4ED fragments in HUVECs.
