# Supplementary material for: Thrombin-cleaved syndecan-3/-4 ectodomain fragments mediate endothelial barrier dysfunction
Source: PLoS One. 2019 May 15;14(5):e0214737. doi: 10.1371/journal.pone.0214737 (PMC6519803; doi:10.1371/journal.pone.0214737)
Supplement: S3 Fig — HUVECs at passage 4 were seeded at 100% confluency onto gelatin-coated electric cell-substrate impedance sensing (ECIS) arrays (8W10E+) (Applied Biophysics, NY, USA) and used in experiments when cell monolayers were measuring a resistance of approximately 1800–2400 ohms. HUVECs were treated with S3ED or S4ED fragments at 100, 300, 1000 or 2000 ng/ml and their TER response measured. (DOCX) [file pone.0214737.s003.docx]

**S3 Fig:**

Concentration-response of S3ED and S4ED fragments on transendothelial electrical resistance (TER) in HUVECs.
