# Supplementary material for: Thrombin-cleaved syndecan-3/-4 ectodomain fragments mediate endothelial barrier dysfunction
Source: PLoS One. 2019 May 15;14(5):e0214737. doi: 10.1371/journal.pone.0214737 (PMC6519803; doi:10.1371/journal.pone.0214737)
Supplement: S4 Fig — HUVECs at passage 4 were seeded at 100% confluency onto gelatin-coated electric cell-substrate impedance sensing (ECIS) arrays (8W10E+) (Applied Biophysics, NY, USA) and used in experiments when cell monolayers were measuring a resistance of approximately 1800–2400 ohms. S3ED or S4ED (100 μg/ml) were incubated with MMP2, MMP9 or MMP14 (5 μg/ml) for 2hr at 37°C. These mixtures were then used to treat the HUVECs on the ECIS arrays with final concentrations of 1 μg/ml SDC ectodomain and 50 ng/ml MMP, and the subsequent TER response was recorded and analyzed. (DOCX) [file pone.0214737.s004.docx]

**S4 Fig:**

MMP (2, 9, 14) treated syndecan-3 and syndecan-4 ectodomains do not affect transendothelial electrical resistance (TER) in HUVECs.
