# Supplementary material for: Thrombin-cleaved syndecan-3/-4 ectodomain fragments mediate endothelial barrier dysfunction
Source: PLoS One. 2019 May 15;14(5):e0214737. doi: 10.1371/journal.pone.0214737 (PMC6519803; doi:10.1371/journal.pone.0214737)
Supplement: S1 Table — List of reagents used in study, including supplier name and catalog number. (DOCX) [file pone.0214737.s005.docx]

**Supporting Table 1:**

List of reagents used in study, including supplier name and catalog number.

| Reagent | Supplier | Catalog number |
| --- | --- | --- |
| 4×Protein Loading Buffer | Li-Cor | 928-40004 |
| Alcian Blue 8GX | MilliporeSigma | A5268 |
| AF488-conjugated donkey anti-mouse | Thermo Fisher Scientific | A21202 |
| AF647-conjugated goat anti-Armenian hamster | Abcam | Ab173004 |
| AF647-conjugated phalloidin | Thermo Fisher Scientific | A22287 |
| Anti-CD31/PECAM-1 (Armenian hamster) | Abcam | ab119341 |
| Anti-syndecan-1 (mouse) | Abcam | ab34164 |
| Anti-syndecan-2 (mouse) | Santa Cruz Biotechnology | sc-365624 |
| Anti-syndecan-3 (mouse) | Santa Cruz Biotechnology | sc-398194 |
| Anti-syndecan-4 (mouse) | Santa Cruz Biotechnology | sc-12766 |
| Anti-VE-Cadherin | Cell Signaling Technology | D87F2 |
| Anti-6-his tag | R & D Systems | MAB050 |
| Bovine Serum Albumin | Fisher | BP1600-100 |
| DAPI hydrochloride | Thermo Fisher Scientific | D1306 |
| DMSO | MilliporeSigma | D2438 |
| Donkey anti-mouse 680 | Li-Cor Biotechnology | 925-68072 |
| Dulbecco's Modified Eagle's Medium | MilliporeSigma | D5796 |
| ELISA S1ED | Abcam | ab47352 |
| ELISA S2ED | LSBio | LS-F12573 |
| ELISA S3ED | R & D Systems | DY3539 |
| ELISA S4ED | R & D Systems | DY2918 |
| Endothelial cell growth media kit | PromoCell | C-22110 |
| Evans Blue | MilliporeSigma | E2129 |
| Formamide | MilliporeSigma | F9037 |
| Glutaraldehyde | Electron Microscopy Sciences | 16010 |
| HUVECs | PromoCell | C-12203 |
| Lactated Ringer's Injection, USP | Hospira | 0409-7953-03 |
| Lanthanum Nitrate Hexahydrate | Electron Microscopy Sciences | 17300 |
| Neutral buffered formalin | Thermo Fisher Scientific | 032-059 |
| Odyssey blocking buffer PBS | Li-Cor Biotechnology | 927-40000 |
| Odyssey Protein Molecular Weight Marker | Li-Cor Biotechnology | 928-40000 |
| ProLong Diamond Antifade Mountant | Thermo Fisher Scientific | P36961 |
| Recombinant human syndecan-1 ectodomain | R & D Systems | 2780-SD050 |
| Recombinant human syndecan-2 ectodomain | R & D Systems | 2965-SD-050 |
| Recombinant human syndecan-3 ectodomain | R & D Systems | 3539-SD-050 |
| Recombinant human syndecan-4 ectodomain | R & D Systems | 2918-SD-050 |
| Recombinant human syndecan-4 ectodomain (*E. Coli*) | Abcam | Ab117191 |
| Recombinant mouse syndecan-3 ectodomain | R & D Systems | 2734-SD-050 |
| Recombinant mouse syndecan-4 ectodomain | R & D Systems | 6267-SD-050 |
| RIPA | EMD Millipore | 20-188 |
| RWJ56110 | Tocris | 2614 |
| SimplyBlue SafeStain | Thermo Fisher Scientific | LC6060 |
| Sodium Cacodylate | Electron Microscopy Sciences | 11653 |
| Static preservation solution (SPS-1)/UW | Organ recovery systems | N/A |
| Thrombin from bovine plasma | MilliporeSigma | T7513 |
| Urethane | MilliporeSigma | 94300 |
| Y27632 | Cayman Chemical | 10005583 |
